# Acoustic and visual adaptations to predation risk: a predator affects communication in vocal female fish

**DOI:** 10.1093/cz/zoab049

**Published:** 2021-06-19

**Authors:** Isabelle Pia Maiditsch, Friedrich Ladich

**Affiliations:** Department of Behavioral and Cognitive Biology, University of Vienna, Althanstraße 14, Vienna 1090, Austria; Department of Behavioral and Cognitive Biology, University of Vienna, Althanstraße 14, Vienna 1090, Austria

**Keywords:** agonistic interactions, antipredator behavior, croaking gouramis, predator inspection, signaling behavior

## Abstract

Predation is an important ecological constraint that influences communication in animals. Fish respond to predators by adjusting their visual signaling behavior, but the responses in calling behavior in the presence of a visually detected predator are largely unknown. We hypothesize that fish will reduce visual and acoustic signaling including sound levels and avoid escalating fights in the presence of a predator. To test this we investigated dyadic contests in female croaking gouramis (*Trichopsis vittata*, Osphronemidae) in the presence and absence of a predator (*Astronotus ocellatus*, Cichlidae) in an adjoining tank. Agonistic behavior in *T. vittata* consists of lateral (visual) displays, antiparallel circling, and production of croaking sounds and may escalate to frontal displays. We analyzed the number and duration of lateral display bouts, the number, duration, sound pressure level, and dominant frequency of croaking sounds as well as contest outcomes. The number and duration of lateral displays decreased significantly in predator when compared with no-predator trials. Total number of sounds per contest dropped in parallel but no significant changes were observed in sound characteristics. In the presence of a predator, dyadic contests were decided or terminated during lateral displays and never escalated to frontal displays. The gouramis showed approaching behavior toward the predator between lateral displays. This is the first study supporting the hypothesis that predators reduce visual and acoustic signaling in a vocal fish. Sound properties, in contrast, did not change. Decreased signaling and the lack of escalating contests reduce the fish’s conspicuousness and thus predation threat.

The ability to communicate effectively with conspecifics and heterospecifics plays a major role in the lives of all animals (Gillam 2011). Animals communicate under ecological constraints which may hinder signal transmission and could impose risks of being detected (Ladich 2019). Predation constitutes one of the main challenges in an animal’s life; it is an important driver of habitat and territory use as well as foraging- and other behavior in all species (Bessey and Heithaus 2013). Animals can be affected by predators in more ways than just by being attacked; different defensive mechanisms as well as behavioral and physiological stress responses evolved in order to increase the chances of survival. Highly vocal taxa such as birds or cetaceans show clear responses as well as adaptations in their vocal as well as social behavior when confronted with predators. Several bird species reduce singing (Krams 2001; Magrath et al. 2010; Schmidt and Belinsky 2013) and fighting behavior when predators appear (Jakobsson et al. 1995). Whales such as sperm whales, gray whales, and belugas responded to playbacks of killer whale calls by changing their social behavior as well as by reducing foraging and sound production (Cummings and Thompson 1971; Fish and Vania 1971; Curé et al. 2013). 

Many studies have documented the effect of predators on fishes. They have demonstrated that fishes can learn to avoid dangerous foraging patches, change their activity patterns, or adapt their behavior, which are effective ways to reduce the risk of predation (Brown et al. 2011; Kelley and Magurran 2011). In shoaling fish, high-predator-density habitats affect the social dynamics and individual’s social interaction (Herbert-Read et al. 2017; Ioannou et al. 2017). Chivers and Smith (1998) listed short-term behavioral responses of different prey fish to chemical alarm signals, including freezing behavior and decreased foraging. Other studies investigated reduced food consumption in the presence of visually detected aerial and aquatic predators. Three-spined stickleback (Milinski 1993, 1985) as well as guppies (Dugatkin and Godin 1992) possess an array of antipredator behaviors to balance feeding and predation risk. The cichlid *Neolamprologus pulcher* and the black carp *Mylopharyngodon piceus* (Fischer et al. 2017; Tang et al. 2017) increased their vigilance and distance to a visually detected predator. Zebrafish *Danio rerio* eavesdrop on the behavior of conspecifics visually exposed to a predator (oscar, *Astronotus ocellatus*) and subsequently display antipredator defensive behaviors (Oliveira et al. 2017). Additional studies on zebrafish investigated habituation to predators and different responses to different types of predators including robotic replicas of oscars (Dugatkin et al. 2005; Bass and Gerlai 2008; Spinello et al. 2019). These studies describe anti-predator behavior of a single or a group of fish during commonplace behavior.

Other studies clarify if and how fish adapt their social interaction and intraspecific signaling (territorial, agonistic, and courtship) when facing a predator. Studies on guppies showed that bolder and shyer guppies exhibited different predator avoidance responses after several exposures (Brown et al. 2018), and male guppies switched from visual signaling during courtship to sneaking when predation risk increases (Endler 1987; Magurran and Seghers 1990). Male guppies preferred courtship over forced mating in the presence of chemical alarm cues potentially benefiting from female preference for bolder males. Female guppies preferred bolder males, but chemical alarm cues trade-off mating and foraging behavior for antipredator behavior in both sexes (Chuard et al. 2020). In the cichlid *Pelvicachromis taeniatus*, a high predation risk during development contributes to maintaining variation in mating preferences and sexual traits (Meuthen et al. 2019). In juvenile convict cichlids *Archocentrus nigrofasciatus*, predation risk caused a decrease in aggression and in size variation on small, but not large foraging patches (Kim et al. 2004). A visually detected predator model modified fighting behavior and visual communication in the goldeneye cichlid *Nannacara anomala* (Jakobsson et al. 1995; Brick 1998). Male sticklebacks performed fewer courtship displays when they received olfactory cues of predator-exposed females (Dellinger et al. 2018), and a juvenile coho salmon *Oncorhynchus kisutch* decreased its aggressive behavior toward a mirror image in the presence of chemical stimuli of an avian predator (Martel and Dill 1993). Weakly electric fishes tended to communicate in a less risky way by reducing amplitudes of low-frequency electric signals in the presence of predators (Stoddard et al. 2019).

In vocal fish which signal acoustically during territorial, agonistic, or courtship behavior, little is known about how predators affect acoustic signaling. A few studies indicate that predators forage by passive listening. Barros and Myrberg (1987) analyzed the stomach contents of bottlenose dolphins *Tursiops truncatus* and noted that they caught numerous sound-producing species (e.g., croakers, toadfishes, and mullets). This observation was substantiated for other predators, which turn toward fish sounds (Gannon et al. 2005; Holt and Johnston 2009). Sound production could increase predation risk by attracting predators and prey fish should therefore respond accordingly when detecting a predator. Silver perch *Bardiella chrysoura*, for example, responded to playbacks of bottlenose dolphin sounds by reducing their calling behavior, as did longspine squirrelfish *Holocentrus rufus* and the Gulf toadfish *Opsanus beta* (Luczkovich et al. 2000; Remage-Healey et al. 2006; Luczkovich and Keusenkothen 2007). Luczkovich et al. (2000) hypothesized that fish showed “acoustic avoidance” by reducing calling behavior. These field experiments, however, lack behavioral observations and thus fail to clarify if the decrease of prey fish calling behavior is accompanied by increased visual signaling, or if fish simply hid in their nest when a predator is present. The present study addresses how vocal fish modify visual and acoustic signaling to maintain intraspecific communication and at the same time reduce conspicuousness and subsequently their predation risk in the presence of a predator.

Our study was designed to investigate the following predictions: 1) agonistic behavior, visual and acoustic signaling decrease in the presence of a predator under standardized conditions, 2) sound characteristics, in particular sound pressure level, decrease in the presence of a predator, and 3) fights end more frequently in the non-escalating phase (lateral display phase) to make the sender less conspicuous when a predator is present. The croaking gourami, *Trichopsis vittata*, was chosen as a model species. Their acoustic and visual signaling during agonistic and reproductive behavior is well studied (Marshall 1966; Ladich et al. 1992a, 1992b; Ladich 1998, 2007; Ladich and Schleinzer 2015). Females were chosen because of availability and because they do not differ from males in signaling during agonistic behavior (Ladich 2007; Ladich and Maiditsch 2018).

## Material and Methods

### Study animals

Twenty-eight female *T. vittata* were investigated during this study (body weight: 1.3–2.4 g; standard length: predator trial 38.3–48.6 mm, and no-predator trial 39.3–48.8 mm), obtained from a local pet supplier. Fish were weighed with a high-accuracy scale (Sartorius GmbH Göttingen PT 120) and measured with a sliding caliper (Workzone, Nr. 23149168). Sexing of fish was based on the presence of the whitish ovary in females, which is visible when holding the fish in a small transparent container against bright light (Supplementary Figure S1). They were kept in community tanks (100 × 50 × 40 cm) at 25 ± 1°C and in a 12-h light–12-h dark cycle. Water was maintained by external filters. Tank bottoms were covered with sand; flowerpots and plants were provided as hiding places. Fish were primarily fed food flakes (Tetramin) 5 times a week.

An oscar *A.* *ocellatus* (Cichlidae) was chosen as a predator model due to its raptorial behavior (Oliveira et al. 2013, 2017). The oscar (body weight: 260 g, standard length: 19.6 cm) was kept singly in a holding tank (100 × 50 × 50 cm) at similar conditions. The tank was equipped with a layer of sand, stones, and artificial plants. The oscar was fed large chironomid larvae, cichlid pellets, or European smelt.

### Experimental design and general information

**Figure 1.**
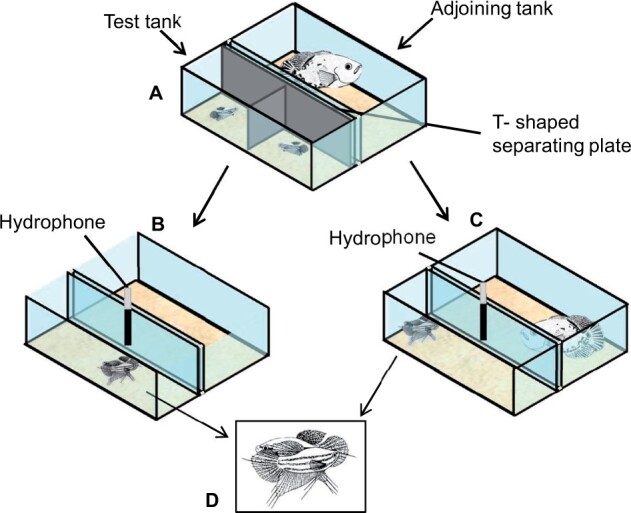
Schematic view of experimental setups. (**A**) All fish were separated from each other by a T-shaped separating plate (gray). Croaking gouramis are visible in the left and right half of the test tank and the oscar in the adjoining tank. (**B**) No-predator and (**C**) predator trial after removal of the separating plate. Both gouramis are displaying laterally (**D**).

Prior to experiments, individual females were kept separately for 5 days in isolation tanks (50 × 27 × 30 cm), under conditions similar to the holding tanks, in order to reduce dominance effects. On the 5th day, fish were transferred individually into the left and right halves of the test tanks (50 × 15 × 30 cm), which were separated by a plastic plate. This plastic plate was non-transparent and T-shaped so that opponents could neither see each other nor the adjoining tank (Figure 1). The test tank bottom was also covered with sand and a plant was placed in each half as a hiding spot. Only gouramis that differed by less than 20% in weight were paired to avoid asymmetries which may not result in a contest (Table 1). Nine predator-present (18 females) and 10 no-predator contests (20 females) were staged. To achieve a higher number of dyadic contests, 10 out of 28 females were used twice, once for a predator as well as once for a no-predator trial, with a 2-week pause between trials to minimize any dominance effects from the first contest. The predator was placed in the adjoining tank (50 × 30 × 30 cm) only during predator experiments. The gap between the 2 aquaria was 0.5 cm wide. *Astronotus ocellatus* was transferred to the adjoining tank a few days before the experiment for adaptation. The feeding of the oscar was suspended for 24 h before the start of the trials to encourage predatory behavior. In every predator trial, the oscar swam rapidly toward the test tank when detecting gouramis, in some cases with spread fins. It also followed the gouramis from one side of the tank to the other, then taking on a stationary, parallel position with spread fins toward the test tank. In a few trials, biting behavior also occurred against the glass toward the gouramis. During no-predator experiments, the adjoining tank remained empty, and the T-shaped plate was placed and lifted in the same way as in the predator trials (Figure 1).

**Table 1.** Mean (±* SE*) asymmetries of body weight and standard length between opponents; 1 indicates no size asymmetry between opponents and behavioral variables of *T. vittata* during predator and no-predator trials

Variable	Predator trials	No-predator trials
	(*N *=* *9)	(*N *=* *10)
Asymmetry of weight	1.08 ± 0.029	1.07 ± 0.018
	(0.96–1.2)	(1–1.15)
Asymmetry of standard length	1.01 ± 0.715	1.02 ± 0.747
	(0.94–1.05)	(0.97–1.07)
Delay until begin of contest (s)	132.8 ± 27.2	109.7 ± 18.5
	(53–224)	(34–203)
Duration of lateral display (s)	13.3 ± 2.07	19 ± 1.88
	(8–22)	(8–27)
Duration of all lateral displays	155.3 ± 50.8	458.3 ± 88.6
(minus pauses, s)	(33–521)	(82–834)
Pauses between lateral displays (s)	119.8 ± 34.5	77.7 ± 10.7
	(15–128)	(32–153)
Number of lateral displays	10.1 ± 1.84	23.5 ± 6.16
	(4–23)	(3–62)
Number of croaking sounds per contest	54.8 ± 23.5	152.7 ± 39.03
	(4–225)	(27–340)
Number of croaking sounds	6.8 ± 1.67	12.2 ± 1.59
per lateral display	(2–14.1)	(3.8–20.3)

*Note*: The range and number of contests analyzed is given in brackets.

The test- and adjoining tank were placed on a table that rested on a vibration-isolated concrete plate. The entire set-up was enclosed in a walk-in semi-anechoic room, which was constructed as a Faraday cage. All experiments were conducted at the same time of the day (around 1,000 h). After experiments, fish were returned to the community tanks. Females that were used twice were returned to the isolation tank and were reused after 2 weeks. They were never paired twice with the same individual or used a second time in the same experimental setup. They were paired with a new opponent because testing the same pair may not result in a contest when a hierarchy has been established during the first fight. Five females started with the predator group and the other 5 were first used in the no-predator trial. After these contests, fish were also returned to the community tanks.

### Behavior and sound recordings

**Figure 2.**
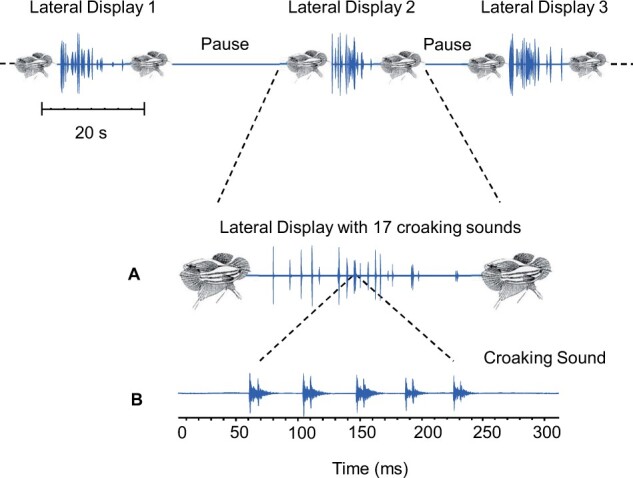
Example of 3 lateral display bouts (1–3) which begin when gouramis spread their unpaired fins, start head-to-tail circling, and alternately produce croaking sounds. Bouts are followed by pauses. (**A**) A lateral display during which 17 croaking sounds were produced and (**B**) oscillogram of 1 sound which consists of 5 double-pulsed bursts (see Ladich and Maiditsch [2018] and Ladich and Schleinzer [2015] for description of vocalizations).

Agonistic behavior and signaling in croaking gouramis consist of 2 phases, the lateral display phase and the frontal display phase. Both phases are organized in bouts (sequences) between which fish paused and usually swam to the water surface for air breathing (Figure 2). The lateral display phase includes visual (lateral) and acoustic display, during which opponents erect their unpaired fins, show head-to-tail circling, and produce croaking sounds (non-escalated phase). The lateral display bouts are followed by frontal displays during which fish protrude their mouths but do not vocalize (escalated phase) (Ladich 1998).

Acoustic signals produced during agonistic interactions consist of a series of bursts. Each burst is produced by 1 pectoral fin, when enlarged fin tendons snap over bony elevations of fin rays (Kratochvil 1978). The dyadic contests started after the fish detected each other visually. Typically, fish emitted sound alternately (in contrast to visual displays which were produced simultaneously) and the sound-producing fish could be recognized by the rapid pectoral fin beating during which the whole animal shook.

Contests were decided 1) during the lateral display phase, when 1 fish gave up and fled (1 winner) or 2) immediately after fish protruded their mouths toward each other. This behavior indicates the beginning of the frontal display phase. Contests which proceeded to the frontal display phase were then stopped by the experimenter to prevent fish from biting each other (Ladich 1998). For convenience, this outcome will be called frontal display phase (outcome: undetermined). Finally, 3) contests could end by termination by both fish during the lateral display phase without a clear winner or loser (outcome: undetermined).

Acoustic signals and behavior were recorded using a hydrophone (Brüel and Kjaer 8101, sensitivity: −186 dB re 1 V/μPa) connected to a microphone power supply (Brüel and Kjaer 2804) which was connected to the XLR mic input of a 4-K video camera (Panasonic HC-X1000). Recordings were operated via the camera display and a video monitor (Sony PVM 4000). The entire setup was positioned behind a curtain so that animals could not see the experimenter.

### Behavioral analysis

The behavior was coded in Sony Vegas Pro 13.0. The following behavioral variables were determined per individual and contests:

The delay until the beginning of a contest (time from removing the separating plate until begin of first lateral display), number and duration of lateral displays in a contest (Figure 2), mean duration of lateral displays (displays start when gouramis spread their unpaired fins and produce croaking sounds and ends when they stop this behavior), duration of all lateral displays (lateral display phase minus pauses), and the duration of pauses between the lateral displays. The number of different types of outcomes was determined. Finally, the number of all approaches to the predator by both fish during a predator contest was determined. Approaching behavior constitutes turning or moving toward the predator in the adjoining tank. Every turn toward the predator was counted as one approach regardless of the duration of approaching behavior. The number of approaches per minute of the lateral display phase was calculated.

### Sound analysis

The video camera recorded LPCM-coded sounds, which were afterward rendered in Sony

Vegas Pro 13.0 to WAV-format (44.1 kHz, 16 bit). These sounds were analyzed using CoolEdit 2000 (Syntrillium Software Corporation, Phoenix, AZ, USA) and S_TOOLS-STX 3.7.8 (Acoustics Research Institute, Austrian Academy of Sciences, Vienna, Austria).

The following sound characteristics were determined for each contest and for each individual: total number of croaking sounds produced during a contest and during each lateral display bout, number of croaking sounds produced per individual, the number of bursts within each croaking sound (sound length) (Figure 2B), the sound pressure level (SPL, LAFmax, at a distance of 5 cm), and the dominant frequency of sounds. The dominant frequency was determined for up to 10 sounds per fish, whereas all other characteristics were determined for all sounds emitted by an individual during a contest.

**Figure 3.**
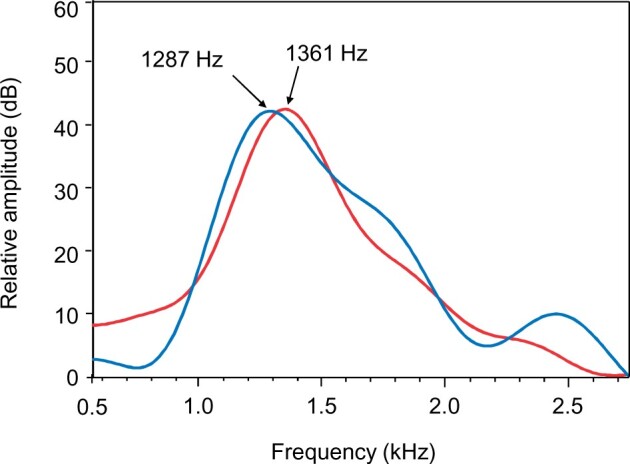
Cepstrum-smoothed power spectra of 2 sound examples produced during a no-predator (blue) and predator (red) trial. Arrows indicate the dominant frequency of these 2 sounds (sampling frequency 48 kHz, filter bandwidth 50 Hz, 75% overlap, number of coefficients: 50, Hanning window).

The dominant frequency of calls was measured using the frequency at the highest spectral level in cepstrum-smoothed power spectra (Figure 3, filter bandwidth 50 Hz, overlap 75%, Hanning window, number of coefficients: 40–50, max. frequency 3.5 kHz) (Noll 1967; Ladich 2007). Frequencies were not analyzed above 3.5 kHz to avoid the resonance frequencies of the small tank (which are above 3.3 kHz according to Akamatsu et al. 2002) and because fish are insensitive to frequencies above 3.5 kHz (Ladich and Yan 1998).

### Sound pressure level measurements

Sound pressure levels (LAFmax, broadband A frequency weighting, RMS Fast time weighting) were recorded using a sound-level meter (Brüel and Kjaer 2250) connected to the microphone power supply. The equipment was calibrated using the hydrophone calibrator (Brüel and Kjaer 4229). Sound pressure levels for each sound were determined using the Measurement Partner Suite (Brüel and Kjær BZ 5503). Because of changing distances of the fish to the hydrophone, the test tank was divided into 50 sectors (5 × 5 cm) by a grid plotted to the front glass of the aquarium. To compensate for different distances between the hydrophone and the vocalizing fish, a correction factor was calculated (Ladich et al. 1992; Ladich 2007; Ladich and Schleinzer 2020). Therefore, a typical croak was played back at a constant level from a small loudspeaker (Fuji 7G06) in each of the 50 sectors. The SPL differences between the sector nearest to the hydrophone (5 cm away) and all other sectors were calculated and added to the SPL values measured, while the fish produced sounds in a particular sector. This yielded a distance-independent absolute SPL for each sound emission (Ladich and Maiditsch 2018).

### Statistical analysis

Behavior variables and sounds emitted during agonistic interactions of a total of 28 croaking gouramis—during 9 predator and 10 no-predator trials—were analyzed. A total of 6 behavior variables and 5 sound characteristics were analyzed; as this is one of the first studies in this direction, we strove to take into account all essential behaviors and vocalizations individually. To control for effects of repeated measurements (individuals used twice), behavioral variables were analyzed using (generalized) linear mixed models in R 4.0.3 (RStudio Team 2020) and additional libraries “nlme” (Pinheiro et al. 2020) and “lme4” (Bates et al. 2015). Models included group (predator, no predator), individual use (once, twice) or order of use (predator–no predator, no predator–predator), and their respective interaction as fixed effects, and individual as random effect to correct for repeated measurements. The repeated use of individuals or their order had no effects whatsoever in these analyses; we therefore omitted these parameters (fixed effects: use and order) and only the fixed effect group, correct for repeated measurements based on the random effect individual, is presented in the “Results” section (Supplementary Tables S1 and S2). Data were tested for normal distribution using the Shapiro–Wilk test. Differences in contest outcomes were tested with Chi-square test (using SPSS 26; IBM SPSS Statistics). Size asymmetries, as well as weight and standard length differences between opponents in predator and no-predator trials, were compared using a *t*-test (using SPSS 26).

The entire contest was analyzed regardless of contest length or number of sounds. Means of behavioral and acoustic variables were calculated for contests and for each individual for both trials and used for further analysis. Agonistic sounds of 10 females in predator trials and 15 females in no-predator trials were analyzed. The remaining fish did not produce sounds during dyadic contests. Only sounds recorded in the first contest of an individual were used for sound analyses.

#### Ethical considerations

Agonistic interactions between croaking gouramis consist of 2 phases: a lateral display phase followed by a frontal display phase. Croaking gouramis produce visual and acoustic signals only during the lateral display phase, without any physical contact between opponents (Ladich 1998). As the intention was to analyze signaling during contests, the agonistic interactions were stopped when contests proceeded to the frontal display phase during which fish bite each other. The predator was kept in the adjacent tank and could not harm test fish. All applicable national and institutional guidelines for the care and use of animals were followed (permit numbers BMWFW-66.006/0035-WF/V/3b/2017; Animal Ethic and Experimental Board, Faculty of Life Science 2017-010).

## Results

### Dyadic contests

No size asymmetries between opponents were found in any trial (Table 1; weight asymmetry: *t*-Test: *t =* −0.409; *df* *=* 17; *P = *0.688; asymmetry of standard length: *t*-Test: *t =* −0.065; *df* *=* 10; *P = *0.528). Also, the weight and standard length did not differ between predator and no-predator trials (weight: *t*-Test: *t =* −0.622; *df* *=* 36; *P = *0.538; standard length: *t*-Test: *t =* −0.267; *df* *=* 36; *P = *0.791). In the presence of the predator, *T. vittata* modified dyadic contests and visual as well as acoustic signaling (Table 1). Agonistic interactions did not proceed to the frontal display phase and thus did not escalate during predator experiments.

**Figure 4.**
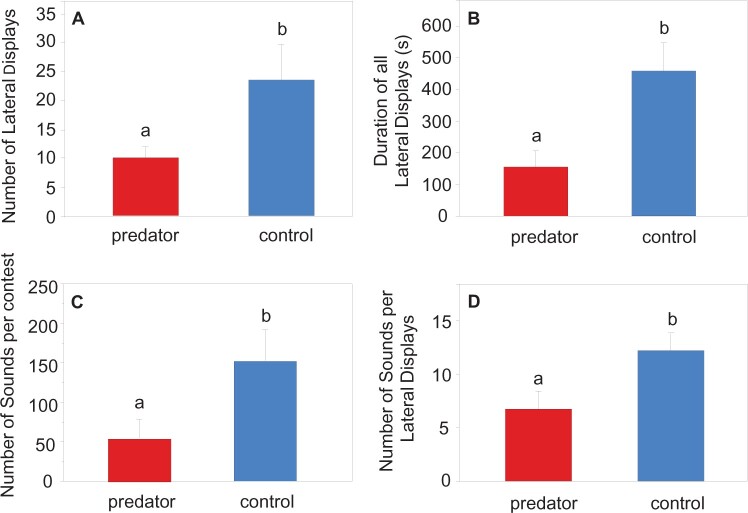
Mean (+SE) behavioral and acoustic variables of *T. vittata* during predator (*N *=* *9) and no-predator (*N *=* *10) trials. (**A**) Number of lateral displays, (**B**) duration of all lateral displays in predator and no-predator experiments, (**C**) number of sounds produced during lateral displays, and (**D**) total number of sounds produced during the entire contest at different trials. Different letters above bars indicate significant differences between experiments (*P < *0.05).

The mean duration of lateral display bouts was shorter during the predator than the no-predator experiments (*F*_1,__9_ = 7.312; *P = *0.014). The number of lateral display bouts per contest decreased significantly in the presence of the predator. An average of 10 lateral display bouts were observed during the predator experiments, but more than twice as many during no-predator experiments (*F*_1,__9_ = 7.3125; *P = *0.024) (Table 1 and Figure 4A). The total duration of all lateral display bouts (minus pauses) was higher in no-predator than predator trials (*F*_1,__9_ = 20.4047; *P = *0.002) (Figure 4B). There was no difference in the delay until the beginning of contests (*F*_1,__9_ = 0.830; *P = *0.385) or in the duration of pauses between lateral displays (*F*_1,__9_ = 1.153; *P = *0.311) (Table 1).

The total number of croaking sounds produced during a dyadic contest was nearly 3 times higher in the no-predator than predator trials (*F*_1,__9_ = 11.7086; *P = *0.007) (Table 1 and Figure 4C). The number of croaking sounds produced per lateral display bout was approximately twice as high in the no-predator versus predator treatment (*F*_1,__9_ = 9.243; *P = *0.014) (Table 1 and Figure 4D).

### Acoustic signals

**Figure 5.**
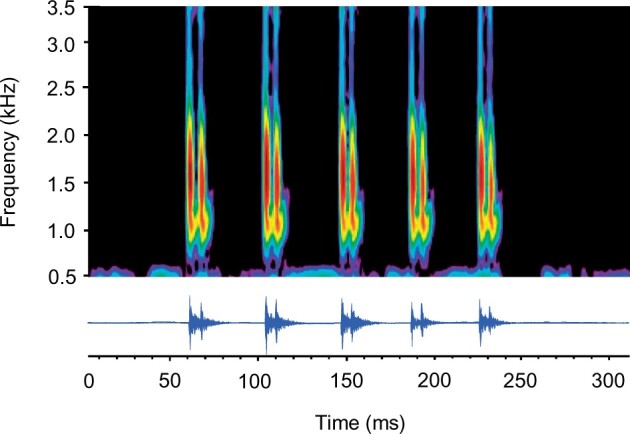
Sonogram (above) and oscillogram (below) of a croaking sound of *Trichopsis vittata*, consisting of 5 double-pulsed bursts. Main energies are between 1 and 2 kHz, pictured by the dark red color which shows the highest energy level (sampling frequency 44.1 kHz, filter bandwidth 250 Hz, 75% overlap, Hanning window).

Croaking sounds produced by *T. vittata* during contests consisted of series of bursts (each one produced by 1 pectoral fin) which were typically built up of 2 pulses (Figure 5). The total number of sounds produced by individual fish during entire contests was twice as high in no-predator versus predator trials (*F*_1,__26_ = 6.227; *P = *0.019) (Table 2). No difference was found in the number of bursts per croaking sound produced in different trials (*F*_1,__26_ = 2.702; *P = *0.112). Neither SPL nor dominant frequencies differed between predator and no-predator trials (SPL: *F*_1,__26_ = 0.12; *P = *0.727; dominant frequency: *F*_1,__26_ = 1.025; *P = *0.321) (Table 2).

**Table 2.** Mean (± *SE*) body weight and sound characteristics of individual *T. vittata* in predator and no-predator trials

Variable	Predator trials	No-predator trials
	(*N *=* *10)	(*N *=* *15)
Body weight (g)	1.8 ± 0.38	1.8 ± 0.08
	(1.3–2.4)	(1.3–2.4)
Number of sounds per individual	36.6 ± 13.76	73.1 ± 16.04
	(2–121)	(6–177)
Number of bursts	4.1 ± 0.22	4.2 ± 0.31
	(2.8–5)	(1.6–5.8)
Dominant frequency (Hz)	1265 ± 29.53	1216.3 ± 28.06
	(1098–1372)	(1106–1436)
Sound pressure level (dB re 1 µPa)	130 ± 1.21	130.7 ± 0.75
	(124.9–135.6)	(125.1–135.6)

*Note*: The range and number of animals measured is given in brackets.

### Outcome of contests and approaching behavior

The outcome of contests differed between experiments. Forty percent of contests proceeded to the FD phase in the no-predator but none in the predator trials. In contrast, 40% of predator experiments were terminated by the fish, which was never observed in the no-predator group (Chi-square: 62.667; df *=* 6; *P < *0.001). The number of contests ending during the LD phase was similar between the 2 treatment types (Table 3).

**Table 3.** Number of contests which ended during the lateral display phase, frontal display phase, or which were terminated by fish in predator and no-predator trials in *T. vittata*

Outcome	Predator trials	No-predator trials
	(*N *=* *9)	(*N *=* *10)
Lateral display phases	5	6
Frontal display phases	0	4
Termination by fish	4	0

Approaching to the adjoining tank was only observed during predator experiments, on an average of 0.46 ± 0.23 (mean ± SE; range: 0.038–0.854) times per minute. It occurred in all predator trials for all 18 individuals. Gouramis approached the predator on average 8 times during a contest (7.9 ± 5.9; 1–19.9).

## Discussion

Predation as an ecological constraint is a very important driver of territory use and social behavior in various taxa and can have a major influence on prey population sizes as well as on environmental structuring. We determined that a single predator in a neighboring tank reduced signaling during contests in *T. vittata*. As we predicted, a decrease in the number and duration of lateral display bouts was accompanied by a decrease in the number of croaking sounds emitted. These data support the hypothesis that agonistic behavior (lateral displaying and sound production) imposes a risk of being detected by predators. Gouramis responded by reducing conspicuous signaling. Nonetheless, sound levels did not decrease as we predicted. Moreover, no escalating behavior was shown during predator experiments, but approaching behavior toward the predator occurred. This indicates that gouramis were more alert in predator versus no-predator trials.

The experimental setup in our study followed that used in numerous previous studies. Milinski (1993) chose a big cichlid *Tilapia mariae* behind a glass partition when studying the effects of predators on foraging behavior in 3-spined sticklebacks *Gasterosteus aculeatus*. Similarly, Oliveira et al. (2013, 2017) chose an oscar as a predator in a study on zebrafish because of its strong predatory behavior. Alternatively, some studies used models of predators outside the tank (Brick 1998). An artificial predator, however, may not be a good choice in all experiments. Preliminary tests in which we used an electrically moveable largemouth bass *Micropterus salmoides* failed to elicit a clear response by croaking gouramis. Our study using a living oscar as a predator model and croaking gouramis as a model for a vocal fish provides for the first time important information on agonistic behavior, acoustic and visual signaling in the presence of predation threat in vocal fish. These data cannot be collected in the field because *T. vittata* inhabits shallow standing waters with dense vegetation, hindering observation. As we hypothesized, *T. vittata* reduced signaling during intraspecific contests in the presence of a predator but did not stop interacting with conspecifics because gouramis need to maintain their territories. Similarly, the Coho salmon *O. kisutch* decreased their aggressive behavior directed toward the mirror image when the odor of an avian predator was present: the total number of acts, intensity of acts, and time spent was significantly lower (Martel and Dill 1993). Goldeneye cichlid *N. anomala* increasingly paused between fighting sequences and changed the rates of their fighting behaviors. Brick (1998) reported that non-escalating behaviors such as lateral display and tail beating increased whereas escalated fighting behaviors (mouth wrestling) decreased when a predator model approached. This is in contrast to croaking gouramis, which reduced lateral displaying in the presence of the oscar. Nonetheless, the contests in both species did not escalate to the frontal display phase in the presence of a predator. Interestingly, contests in *T. vittata* could end without a clear decision, namely a winner in the present study. This breaking off of agonistic interactions was never described in prior studies (Ladich 1998; Ladich and Schleinzer 2015; Ladich and Maiditsch 2018) and never occurred in the no-predator group of the present study. Typically, dyadic contests escalated when size asymmetry between contestants was small and when it took longer to assess the fighting ability of opponents (Brick 1998; Ladich 1998). To avoid size influencing the outcome of a contest, the size asymmetry was minimized in all trials of the current study. Termination of agonistic interactions in the presence of a predator is apparently another strategy to reduce a predatory threat. Brick (1998) observed that *N. anomala* terminated a fighting bout in order to flee from the approaching predator model. In contrast, croaking gouramis did not flee, but stayed in one half of the test tank without starting the contest again. Clearly, croaking gouramis use several strategies to reduce predation risk. This includes shorter fighting bouts (lateral displays), less visual and acoustic signaling, no escalation to the risky frontal display phase, or even termination of a contest.

Another behavior which we observed only in the predator experiments was approaching the adjoining tank. We interpret this as predator-inspection behavior, which could reduce the risk of being attacked by predators. Inspection or approaching behavior may indicate risk assessment and protect individuals from attacks and therefore yield additional benefits (Godin and Davis 1995).

Ladich (1998) showed that the production of croaking sounds is apparently decisive for the outcome of dyadic contests in *T. vittata.* However, acoustic communication poses an increased risk because predators may detect acoustic signals of vocalizing prey via passive listening (Barros and Myrberg 1987; Lima and Dill 1990). Animals would therefore be expected to adapt their acoustic signaling to reduce predation threat. Croaking gouramis reduced their calling activity when detecting the predator visually. Luczkovich and Keusenkothen (2007) concluded, based on lower vocalization rates during playbacks of dolphin echolocation sounds, that squirrelfish show “acoustic avoidance” behavior. Playbacks of dolphin sounds in the field also resulted in a lower calling rate in the Gulf toadfish (Remage-Healey et al. 2006) and in the silver perch (Luczkovich et al. 2000). The calling activity was clearly predator dependent in *T. vittata*, dropping significantly when a predator was present. This decrease in visual and acoustic signaling during contests indicates that their conspicuousness decreases during agonistic interactions.

Otherwise, croaking gouramis shortened neither the length of their sounds (number of bursts) nor the sound pressure level. We hypothesized that vocal fish will reduce the SPL of acoustic signals to be less conspicuous. This was not confirmed in the current study: sound levels did not differ between no-predator and predator trials. This lack of reduction in intensity in the current study could theoretically indicate that the animals are unable to significantly reduce sound length or sound level because they have only 1 vocal motor pattern within the central nervous system which elicits just 1 pattern of sonic muscle contraction and subsequently 1 type of agonistic sound (Ladich and Bass 2011; Bass et al. 2015). This, however, is not the case in female croaking gouramis, which have a larger vocal repertoire than males and produce 2 types of sounds, namely croaking sounds during agonistic interactions and purring sounds prior to spawning (Marshall 1966; Ladich 2007). Purring sounds have a lower SPL and are shorter than croaking sounds (Ladich 2007). Clearly, fighting over a territory would not benefit from reducing sound length and lowering SPLs. In order to indicate high fighting abilities and avoid losing the contest, they need to produce long and loud sound. In contrast, female purring sounds are lower in level and duration and thus less conspicuous, making courtship and spawning less likely to be detected and interrupted by conspecifics and predators. The dominant frequency of sounds did not differ between experiments. This is probably due to the fact that dominant frequency depends on body size in fish producing pulsed sounds such as croaking gouramis (Ladich et al. 1992; Myrberg et al. 1993; Ladich and Maiditsch 2018). The fish chosen for our experiments were similar in size and thus dominant frequency did not differ between predator and no-predator trials.

In conclusion, the current study is to our knowledge the first to show that 1 strategy of vocal fish is to modify acoustic and visual communication in various ways during social interactions to increase survival in the presence of a single predator fish (oscar). The presence of a predator affects visual and acoustic communication during agonistic contests in a highly vocal fish, the croaking gourami under laboratory conditions. These observations confirm the hypothesis that gouramis reduce the extent of visual and acoustic signaling as well as avoid escalated fighting in the presence of a predator in order to reduce conspicuousness and increase vigilance. Moreover, gouramis do not cease agonistic interactions entirely against intraspecific intruders because defending territories is essential for reproduction. Our results reveal that predators modify agonistic interaction and the way contests end. In addition, the findings show that fights over resources such as territories do not stop in the presence of a predator. Furthermore, croaking gouramis approach and inspect the predator (oscar), which is an additional way of reducing predation risk. What remains to be addressed is the variation in the behavior of a single predator used, which could be a confounding factor that may influence the outcome of contests. Future studies could be done with multiple predators, which can support our findings or show different behavior due to different predators. Importantly, this study shows that predators affect acoustic and visual communication in vocalizing fish species. Playing back predator’s calls via underwater speakers and recording prey fish sounds is not sufficient to describe behavioral adaptation to predation risk in vocal fish species. Although further studies are needed, our data suggest that a visually detected predator is an important ecological constraint modifying social behavior and communication in a highly territorial teleost in its native ecosystem.

## Author Contributions

I.P.M. and F.L.: conceptualization, methodology, and writing. I.P.M.: investigation and analyses. F.L.: resources, supervision, and funding acquisition. 

## Supplementary Material

zoab049_Supplementary_Data
