# Supplementary material for: Acoustic and visual adaptations to predation risk: a predator affects communication in vocal female fish
Source: Curr Zool. 2021 Jun 19;68(2):149–57. doi: 10.1093/cz/zoab049 (PMC8962716; doi:10.1093/cz/zoab049)
Supplement: zoab049_Supplementary_Data [file zoab049_supplementary_data.zip › zoab049-suppl_data/Supplementary Material.docx]

**Supplementary Material**


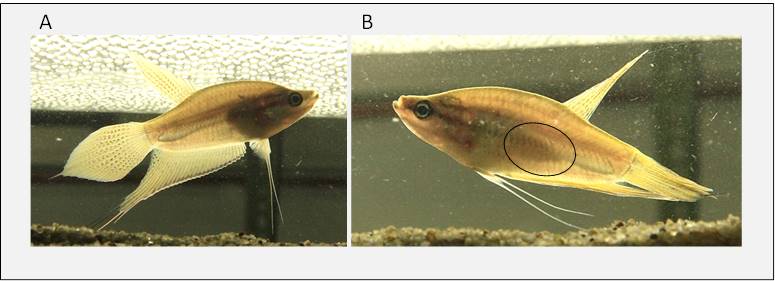


Supplementary Figure 1: Picture of a Male (A) and a female (B) croaking gourami. The black ellipse caudal of the viscera indicates the whitish ovary.
