# Supplementary material for: Acoustic and visual adaptations to predation risk: a predator affects communication in vocal female fish
Source: Curr Zool. 2021 Jun 19;68(2):149–57. doi: 10.1093/cz/zoab049 (PMC8962716; doi:10.1093/cz/zoab049)
Supplement: zoab049_Supplementary_Data [file zoab049_supplementary_data.zip › zoab049-suppl_data/Supplementary Table 1 and Table 2.pdf]

Supplementary Table 1: Full model analyses including trial (predator, no predator), individual use (once, twice) and their respective interaction as fixed effects, and individual as random effect.

Variable		Value	Std.Error	DF	t-value	p-value
**Delay until begin of contest (s)**	Intercept	4.5332	0.1984	26	22.8471	0.0000
	Trial	0.3551	0.2976	8	1.1931	0.2670
	Used	-0.0014	0.2806	26	-0.0050	0.9960
	Trial:Used	-0.3037	0.3948	8	-0.7692	0.4639
**Random factors**	Individual	Residual				
		0.5488				

**Duration of lateral display (s)**	Intercept	2.9211	0.1354	26	21.5627	0.0000
	Trial	-0.2564	0.2032	8	-1.2619	0.2425
	Used	-0.0632	0.1915	26	-0.3299	0.7441
	Trial:Used	-0.2690	0.2773	8	-0.9700	0.3604
**Random factors**	Individual	Residual				
		0.3993				

**Duration of all lateral displays (minus pauses, s)**	Intercept	5.7906	0.2513	26	23.0372	0.0000
	Trial	-0.6084	0.3770	8	0.5174	0.1452
	Used	0.1839	0.3554	26	0.5174	0.6092
	Trial:Used	-1.0919	0.5039	8	-2.1668	0.0621
**Random factors**	Individual	Residual				
		0.7071				

**Pauses between lateral displays (s)**	Intercept	4.0829	0.2020	26	20.2046	0.0000
	Trial	0.6006	0.3031	8	1.9814	0.0829
	Used	0.3738	0.2857	26	1.3082	0.2022
	Trial:Used	-0.7118	0.4143	8	-1.7181	0.1241
**Random factors**	Individual	Residual				
		0.5974				

**Number of lateral displays**	Intercept	2.6862	0.2340	26	11.4758	0.0000
	Trial	-0.1660	0.3511	8	-0.4728	0.6489
	Used	0.2407	0.3310	26	0.7273	0.4735
	Trial:Used	-0.8230	0.4573	8	-1.7994	0.1096
**Random factors**	Individual	Residual				
		0.6200				

**Number of croaking sounds per contest**	Intercept	4.3677	0.3567	26	12.2442	0.0000
	Trial	-0.6887	0.5350	8	-1.2872	0.2340
	Used	0.5010	0.5044	26	0.9931	0.3289
	Trial:Used	-1.1836	0.7353	8	-1.6095	0.1462
**Random factors**	Individual	Residual				
		1.0670				

**Number of croaking sounds per lateral display**	Intercept	2.3447	0.1972	26	11.8854	0.0000
	Trial	-0.4150	0.2959	8	-1.4025	0.1983
	Used	0.1061	0.2789	26	0.3806	0.7006
	Trial:Used	-0.6143	0.4067	8	-15.105	0.1693
**Random factors**	Individual	Residual				
		0.5901				

Supplementary Table 2: Full model analyses including only individuals used twice; trial (predator, no predator), order of use (predator-no predator, no predator-predator) and their respective interaction as fixed effects. Individual as a random effect.

**Variable**		**Value**	**Std.Error**	**DF**	**t-value**	**p-value**
**Delay until begin of contest (s)**	Intercept	4.7736	0.3284	8	14.5316	0.0000
	Trial	-0.2900	0.4027	8	-0.7200	0.4920
	Order	-0.4835	0.4645	8	-1.0408	0.3284
	Trial:Order	0.6827	0.5695	8	1.1987	0.2649
**Random factors**	Individual	Residual				
		0.5695				

**Duration of lateral display (s)**	Intercept	2.7667	0.1931	8	14.3278	0.0000
	Trial	-0.2495	0.2522	8	-0.9890	0.3514
	Order	0.1823	0.2730	8	0.6676	0.5231
	Trial:Order	-0.5518	0.3566	8	-1.5472	0.1604
**Random factors**	Individual	Residual				
		0.3566				

**Duration of all lateral displays (minus pauses, s)**	Intercept	6.2280	0.3012	8	20.6769	0.0000
	Trial	-1.4270	0.4259	8	-3.3501	0.0101
	Order	-0.5069	0.4259	8	-1.1900	0.2681
	Trial:Order	-0.5466	0.6024	8	-0.9074	0.3907
**Random factors**	Individual	Residual				
		0.6024				

**Pauses between lateral displays (s)**	Intercept	4.2458	0.3862	8	10.9933	0.0000
	Trial	0.1995	0.5269	8	0.3786	0.7148
	Order	0.4218	0.5461	8	0.7723	0.4621
	Trial:Order	-0.6216	0.7452	8	-0.8341	0.4284
**Random factors**	Individual	Residual				
		0.7452				

**Number of lateral displays**	Intercept	3.0951	0.2868	8	10.7899	0.0000
	Trial	-0.8067	0.3984	8	-2.0246	0.0775
	Order	-0.3362	0.4056	8	-0.8289	0.4312
	Trial:Order	-0.3646	0.5635	8	-0.6471	0.5357
**Random factors**	Individual	Residual				
		0.5635				

**Number of croaking sounds per contest**	Intercept	4.9236	0.3596	8	13.6905	0.0000
	Trial	-1.3023	0.5086	8	-2.5607	0.0336
	Order	-0.1097	0.5086	8	-0.2157	0.8346
	Trial:Order	-1.1400	0.7192	8	-1.5849	0.1516
**Random factors**	Individual	Residual				
		0.7192				

**Number of croaking sounds per lateral display**	Intercept	2.4623	0.2080	8	11.8357	0.0000
	Trial	-0.7430	0.2942	8	-2.5255	0.0355
	Order	-0.0227	0.2942	8	-0.0774	0.9402
	Trial:Order	-0.5726	0.4160	8	-1.3762	0.2060
**Random factors**	Individual	Residual				
		0.4160				
